# *Callicarpa bachmaensis* Soejima & Tagane (Lamiaceae), a new species from Bach Ma National Park in Thua Thien Hue Province, Central Vietnam

**DOI:** 10.3897/phytokeys.62.7974

**Published:** 2016-03-25

**Authors:** Akiko Soejima, Shuichiro Tagane, Ngoc Nguyen Van, Chinh Nguyen Duy, Nguyen Thi Thanh Huong, Tetsukazu Yahara

**Affiliations:** 1 Department of Biological Sciences, Graduate School of Science and Technology, Kumamoto University, 2-39-1 Kurokami, Kumamoto 860-8555, Japan; 2 Center for Asian Conservation Ecology, Kyushu University, 744 Motooka, Fukuoka, 819-0395, Japan; 3 Department of Biology, Dalat University, 1 – Phu Dong Thien Vuong, Dalat, Vietnam; 4 Dept. of Botany, Institute of Ecology and Biological Resources (IEBR), Vietnam Academy of Science and Technology (VAST), 18 Hoang Quoc Viet Road, Hanoi, Vietnam

**Keywords:** Bach Ma National Park, *Callicarpa*, Lamiaceae, new species, Thua Thien Hue, Vietnam

## Abstract

A new species, *Callicarpa bachmaensis* Soejima & Tagane, **sp. nov.**, is described and illustrated from Bach Ma National Park in Thua Thien Hue Province, Central Vietnam. This species has a characteristic liana habit, white corolla, and narrowly elliptic to narrowly lanceolate, entire, occasionally subequal leaves, by which it is clearly distinguished from the other previously known species of this genus.

## Introduction

The genus *Callicarpa* L. (Lamiaceae) comprises 154 species and 12 varieties (The Plant List 2013) of shrubs or small trees, rarely woody climbers, distributed in temperate and tropical regions. The center of species diversity of this genus is in the Old World, especially in Malesia where about 50 species occur (Bramley 2013). Common and well-known species are found widely in secondary forests or in disturbed areas, but many species are restricted to small areas in primary forest (Bramley 2009, 2013). The genus is characterized by simple, opposite leaves, small white to pink or violet flowers in cymose or thyrsoid infloresences, small globose drupes, and often stellate, plumose, or dendroid hairs on stems, leaves, calyces, and fruits (Chen and Gilbert 1994, Bramley 2009, 2013, Leeratiwong et al. 2009).

The species of Vietnam have been studied by de Loureiro (1790), Dop (1935), Ho (2003), and most recently by Phuong (2007) who enumerated 20 species. During a botanical survey of Bach Ma National Park in Thua Thien Hue Province, Central Vietnam in 2015, we found a species that was different from all the other known species of *Callicarpa*. This plant is described and illustrated as a new species, *Callicarpa bachmaensis* Soejima & Tagane.

## Taxonomy

### Callicarpa bachmaensis

Taxon classificationPlantaeLamialesLamiaceae

Soejima & Tagane sp. nov.

urn:lsid:ipni.org:names:77153917-1

Figs 1 , 2

#### Type.

VIETNAM. Thua Thien Hue Province, Bach Ma National Park, edge of evergreen forest along stream, 16°13'36.70"N, 107°51'07.22"E, alt. 414 m, 24 May 2015, with flowers, *Tagane S., Toyama H., Yahara T., Ngoc Nguyen, Chinh Nguyen, Okabe N. V2677* (holotype KYO!, isotypes BKF!, DLU!, FU!, P!, VNM!, the herbarium of Bach Ma National Park!).

#### Diagnosis.

*Callicarpa bachmaensis* is distinguishable from the other species of *Callicarpa* by a combination of its liana habit, white flowers and narrowly elliptic to narrowly lanceolate, entire, occasionally subequal leaves. *Callicarpa angusta* Schauer, endemic to Philippines, and *Callicarpa angustifolia* King & Gamble, distributed in China, Cambodia, Thailand, Vietnam, and Peninsular Malaysia, resemble to *Callicarpa bachmaensis* in possessing narrowly elliptic leaves. However, they differ from *Callicarpa bachmaensis* in having a shrubby habit and serrate leaves. Additionally, *Callicarpa angusta* differs in having hairy corollas, and *Callicarpa angustifolia* differs in having larger leaves (more than 9 cm long in *Callicarpa angustifolia* vs. less than 9 cm long in *Callicarpa bachmaensis*) and larger corollas (ca. 3 mm long vs. 2–2.2 mm long).

#### Description.

Liana-like small tree, ca. 3 m tall. Branches sweeping with gradual curve and overhanging distally. Twigs with a dense indumentum of short plumose or dendroid brown hairs, slightly 4-angular when young, later becoming terete, woody and glabrous, lenticellate; internode 8.7–13 cm long in main stems, 1.4–3 cm long in axial ones. Leaves opposite, occasionally subequal; blades narrowly elliptic to narrowly lanceolate, (2.6–)5–8 × (0.4–)0.8–2 cm in larger leaves, 1.1–4 × 0.2–1 cm in smaller ones, base acute to cuneate, apex acuminate, margin entire, upper surface with short stellate hairs or sometimes almost glabrous, also with small yellow sessile glands and a few larger sessile glands, lower surface densely covered with white stellate or sometimes pale brownish fringed peltate scale-like hairs and a few larger sessile glands, chartaceous to subcoriaceus, green and slightly lustrous above, white hairy below; midrib slightly sunken above, prominent below, secondary veins 6–10 pairs, prominent below, tertiary veins scalariforming-reticulate; petioles 0.8–1.1 cm long, with indumentum as branches. Inflorescence axillary, cymose, entirely covered with indumentum as branches, peduncles 0.8–1 cm long; bracts linear, 1.5–2 mm long; pedicels ca. 0.5 mm long. Calyx cup-shaped, 1–1.5 mm long with 4 (or 5) shallow lobes, outer surface covered with stellate or short dendroid hairs and small yellow sessile glands, inner surface almost glabrous. Corolla white, 2–2.2 mm long, divided into four lobes, tube ca. 1 mm long, lobes oblong-ovate, <1 mm long, apex rounded, outer surface with yellow sessile glands, inner surface almost glabrous. Stamens 4, ca. 1 mm long exerted from corolla, filaments 2.5–3 mm long, glabrous, anthers elliptic, ca. 1 mm long, with many yellow sessile glands near connectives, dehiscing through longitudinal slits. Stigma capitate, style ca. 6 mm long, glabrous. Fruits drupaceous, spherical, 2.5–3 mm in diameter, dark purple, sparsely covered with stellate hairs.

#### Specimen examined.

Vietnam. 20 June 1976, with fruits, *Ly Quoc Anh 106* (HN!); Lao Cai Province: Liem Phu, Van Ban, 10 June 2008, with flowers, *Binh N.Q. & Cuong D.D. VN1985* (HN!); Quang Nam-Da Nang Province: Hà ra, Giăng village, 13 July 1986, with fruits, *LX-VN 2958* (HN!); Vinh Phuc Province: Me Linh Biodiversity Station, Ngoc Thanh Commune, 24 Oct. 2001, with fruits, *Phuong et al. 4611* (HN!).

#### Phenology.

Flowering specimens were collected in May and June; fruiting in June, July, and October.

#### Distribution and habitat.

VIETNAM: Northeast (Lao Cai and Vinh Phuc Province) and Central coast (Thua Thien Hue, Quang Nam and Da Nang Province) (Fig. 3). In the type locality, Bach Ma National Park, Thua Thien Hue Province, a small population was found at the edge of humid broad-leaved evergreen forest along a stream, at altitude ca. 400 m, in which *Dipterocarpus hasseltii* Blume (Dipterocarpaceae), *Croton argyratus* Blume (Euphorbiaceae), *Vitex axillariflora* (Merr.) Bramley (Lamiaceae), *Beilschmiedia henghsienensis* S. K. Lee & Y. T. Wei (Lauraceae), *Litsea balansae* Lecomte (Lauraceae), *Heritiera augustata* Pierre (Malvaceae), *Syzygium diospyrifolium* (Wall. ex Duthie) S. N. Mitra (Myrtaceae), *Syzygium siamense* (Craib) Chantar. & J. Parn. (Myrtaceae), *Adina pilulifera* (Lam.) Franch. ex Drake (Rubiaceae) dominate.

**Figure 1.**
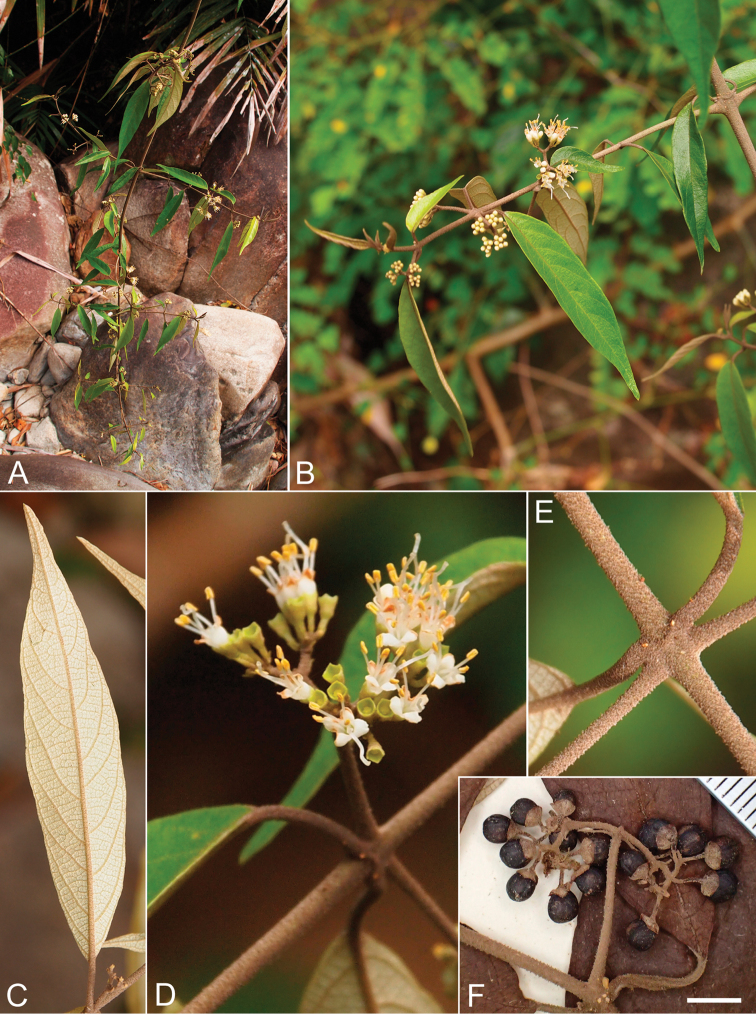
*Callicarpa bachmaensis* Soejima & Tagane, sp. nov. **A** Branch apex **B** Flowering branch **C** Abaxial surface of lamina **D** Inflorescences **E** Twigs and base of petiole, **F** Infructescence [*Binh & Cuong VN1985* (HN). Scale bar E = 5 mm].

**Figure 2.**
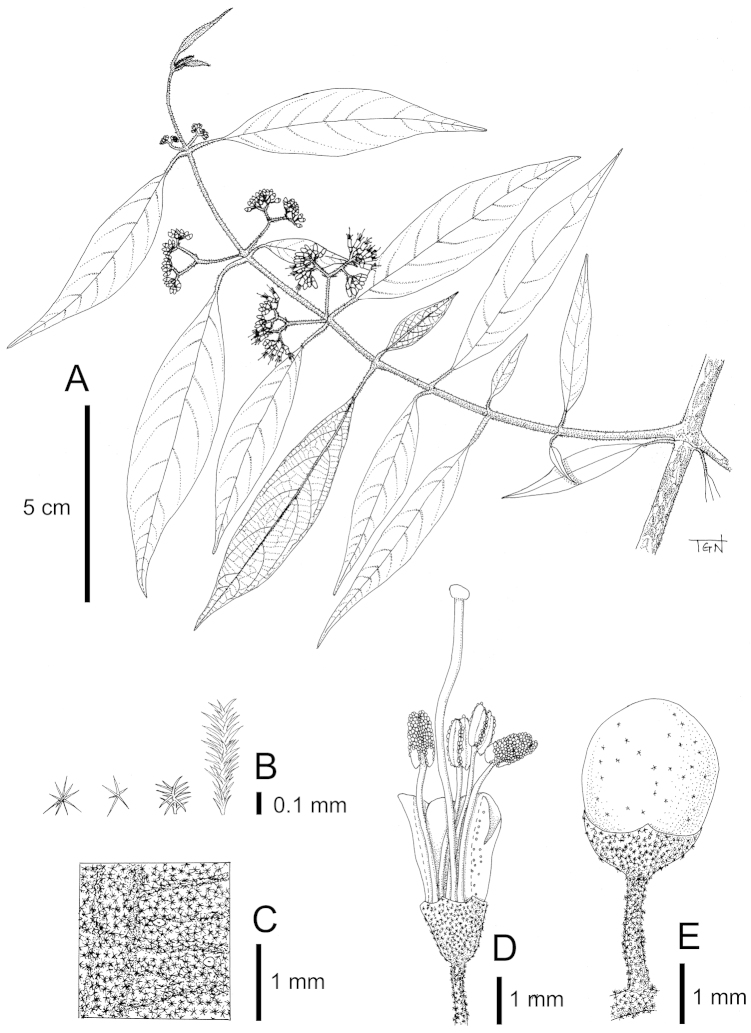
*Callicarpa bachmaensis* Soejima & Tagane, sp. nov. **A** Flowering branch **B** Stellate and dendroid hairs on calyx (left three) and lower surface of leaves (right) **C** Abaxial surface of lamina **D** Flower with the corolla dissected to show filaments and style **E** Fruit. Materials: *Tagane et al. V2677*.

**Figure 3.**
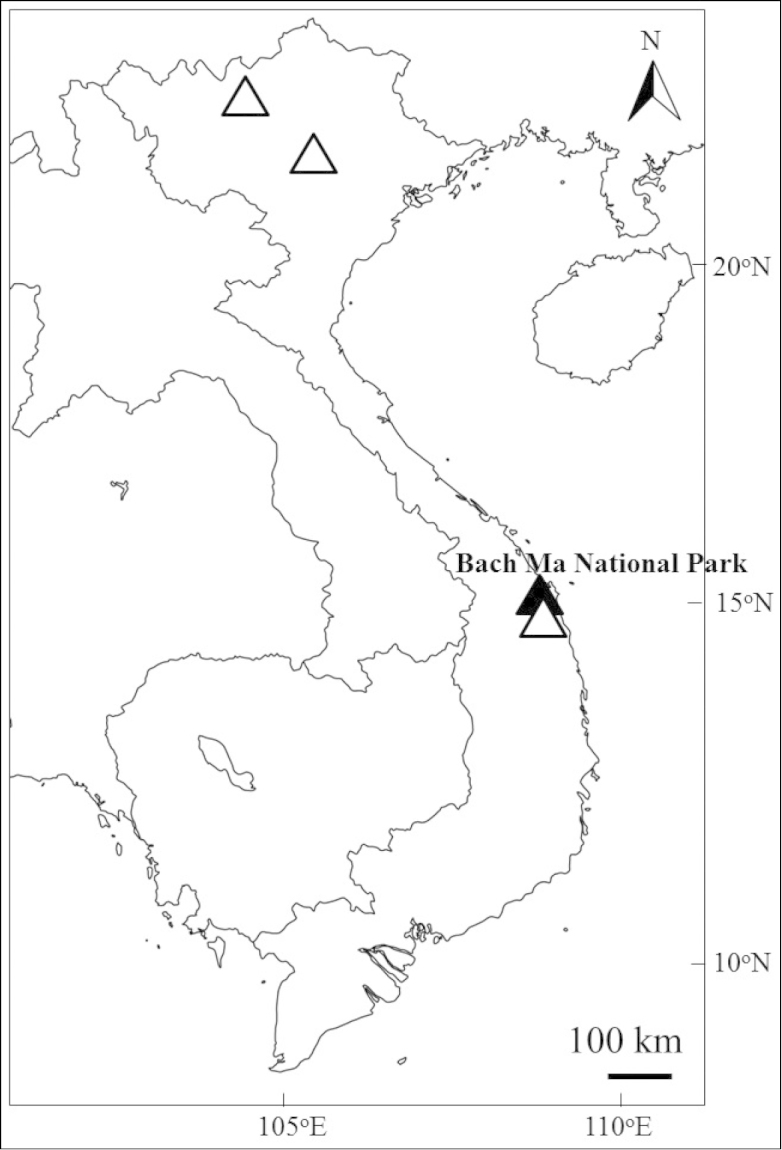
Distribution of *Callicarpa bachmaensis* Soejima & Tagane. Black triangle: type locality in Bach Ma National Park; white triangle: other collection sites in Vietnam.

#### GenBank accession no.

*Tagane et al. V2677*: LC120829 (*rbcL*), LC120830 (*matK*).

#### Etymology.

The specific epithet *bachmaensis* reflects the name of the locality where the type specimen was collected.

#### Conservation status.


 Data Deficient (DD). *Callicarpa bachmaensis* is collected from north and central Vietnam. In the type locality, Bach Ma National Park, the number of individuals is likely stable, but at present we have no reliable information on its abundance and range. Further investigations are needed to accurately assess its status in the natural habitat.

## Supplementary Material

XML Treatment for Callicarpa bachmaensis

## References

[B1] Bramley GLC (2009) The genus *Callicarpa* (Lamiaceae) on Borneo. Botanical Journal of Linnean Society 159: 416–455. doi: 10.1111/j.1095-8339.2009.00907.x

[B2] Bramley GLC (2013) The genus *Callicarpa* (Lamiaceae) in the Philippines. Kew Bulletin 68: 369–418. doi: 10.1007/s12225-013-9456-y

[B3] Briquet J (1897) Verbenaceae. In: Engler A Prantl K (Eds) Die Naturlichen Pflanzenfamilien Teil 4, Abt. 3a W. Engelmann, Leipzig, 132–182. [In German]

[B4] Chang H-T (1951) A review of the Chinese species of *Callicarpa*. Acta Phytotaxonomica Sinica 1: 269–312. [In Chinese]

[B5] Chen S-L Gilbert MG (1994) *Callicarpa*. In: Wu ZY Raven PH (Eds) Flora of China 17: 4–16.

[B6] de Loureiro J (1790) Flora Cochinchinensis Vol. 1. Lissabon, Ulyssipone, 432 pp [In Latin]

[B7] Dop P (1935) Verbenaceae. In: Lecomte H (Ed.) Flore Générale de L’Indochine 4, Paris, 775–913. [In French]

[B8] Ho PH (2003) An Illustrated flora of Vietnam Vol. 2. Young Publishing House, Ho Chi Minh City, 951 pp [In Vietnamese]

[B9] Leeratiwong C Chantaranothai P Paton AJ (2009) A synopsis of the genus *Callicarpa* L. (Lamiaceae) in Thailand. Thai Forest Bulletin (Botany) 37: 36–58.

[B10] Phuong VX (2007) Flora of Vietnam 6 -Verbenaceae. Science & Technics Publishing House, Hanoi, 283 pp.

[B11] The Plant List (2013) Version 1.1 Published on the Internet http://www.theplantlist.org/ [accessed 1st January, 2016]

